# Four cases of disseminated herpes simplex virus following talimogene laherparepvec injections for unresectable metastatic melanoma

**DOI:** 10.1016/j.jdcr.2022.12.023

**Published:** 2023-01-27

**Authors:** Kate E. Beekman, Lily M. Parker, Danielle K. DePalo, Kelly M. Elleson, Amod A. Sarnaik, Kenneth Y. Tsai, Bethany M. Withycombe, Jonathan S. Zager

**Affiliations:** a USF Health, Morsani College of Medicine, Tampa, Florida; b Department of Cutaneous Oncology, Moffitt Cancer Center, Wesley Chapel, Florida; c Department of Anatomic Pathology and Tumor Biology, Moffitt Cancer Center, Wesley Chapel, Florida; d Department of Pharmacy, Moffitt Cancer Center, Wesley Chapel, Florida

**Keywords:** acyclovir, adverse events, adverse reactions, dermatology, disseminated herpes infection, drug eruption, drug-induced reactions, drug reactions, herpes encephalitis, herpes simplex virus 1, IMLYGIC, immunotherapy, intralesional injections, malignant melanoma, melanoma, metastatic melanoma, oncolytic injection, oncolytic virus, regional chemotherapy, regional therapy, skin cancer, surgical oncology, T-VEC, talimogene laherparepvec, valacyclovir, viral dermatitis, viral encephalitis, viral rash

*To the Editor:* In the article titled “Durable melanoma control following disseminated talimogene laherparepvec herpetic infection,” Shmuylovich et al^1^ presented a case of disseminated herpes simplex virus (HSV) following talimogene laherparepvec (T-VEC) intralesional therapy for unresectable locally advanced melanoma. We are aware of 1 other published case by Kimmis et al.^2^ Here, we describe 4 additional cases of disseminated HSV in immunocompetent patients following T-VEC therapy. The patients consented to having nonidentifying photographs taken and utilized for educational and research purposes as well as sharing of descriptions of their cases and nonidentifying information.

## Case 1

**Fig 1:**
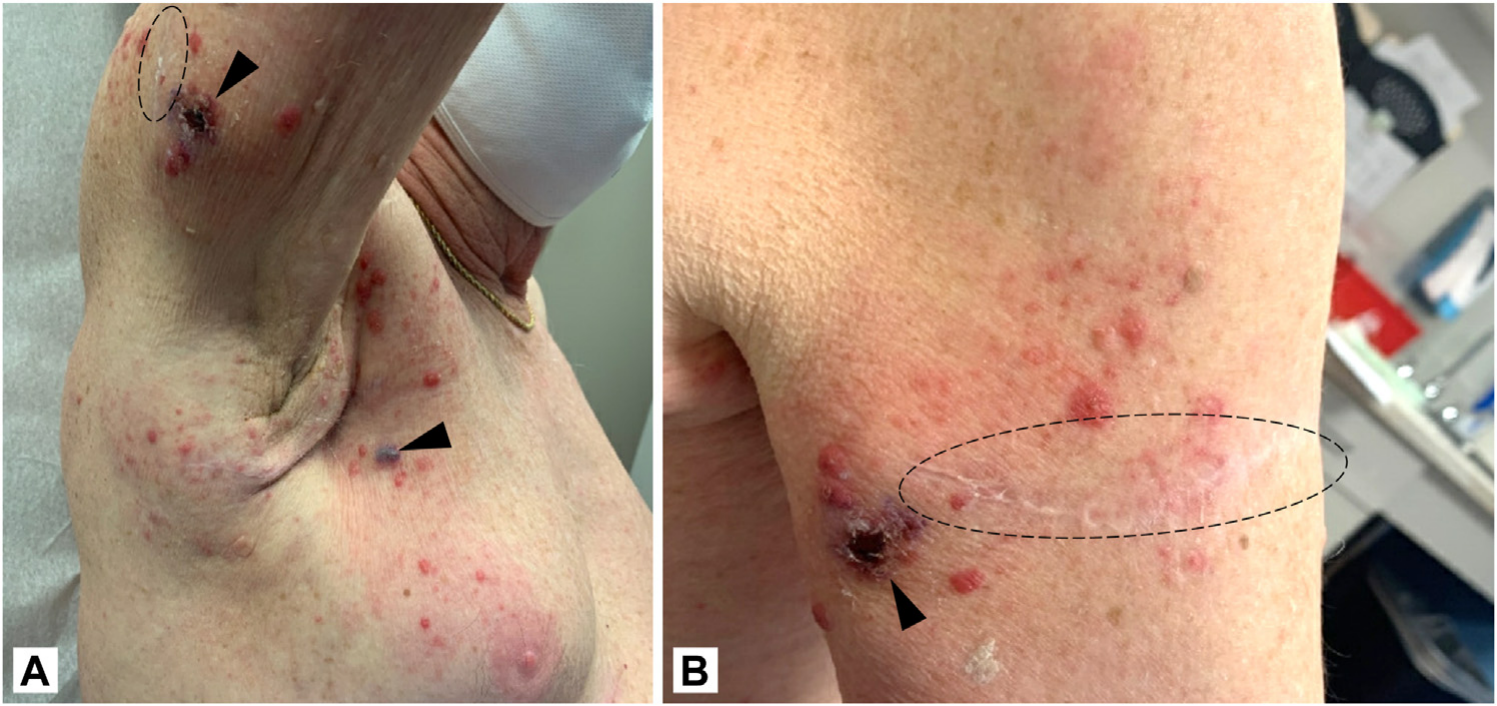
A, B, Case 1. Grouped vesicles on an erythematous base across the axilla and posterior aspect of the arm 10 days after talimogene laherparepvec injection. The *dashed ellipse* encompasses the surgical scar from the excision of the primary melanoma. *Arrowheads* designate the nodules of in-transit melanoma injected with talimogene laherparepvec.

**Fig 2:**
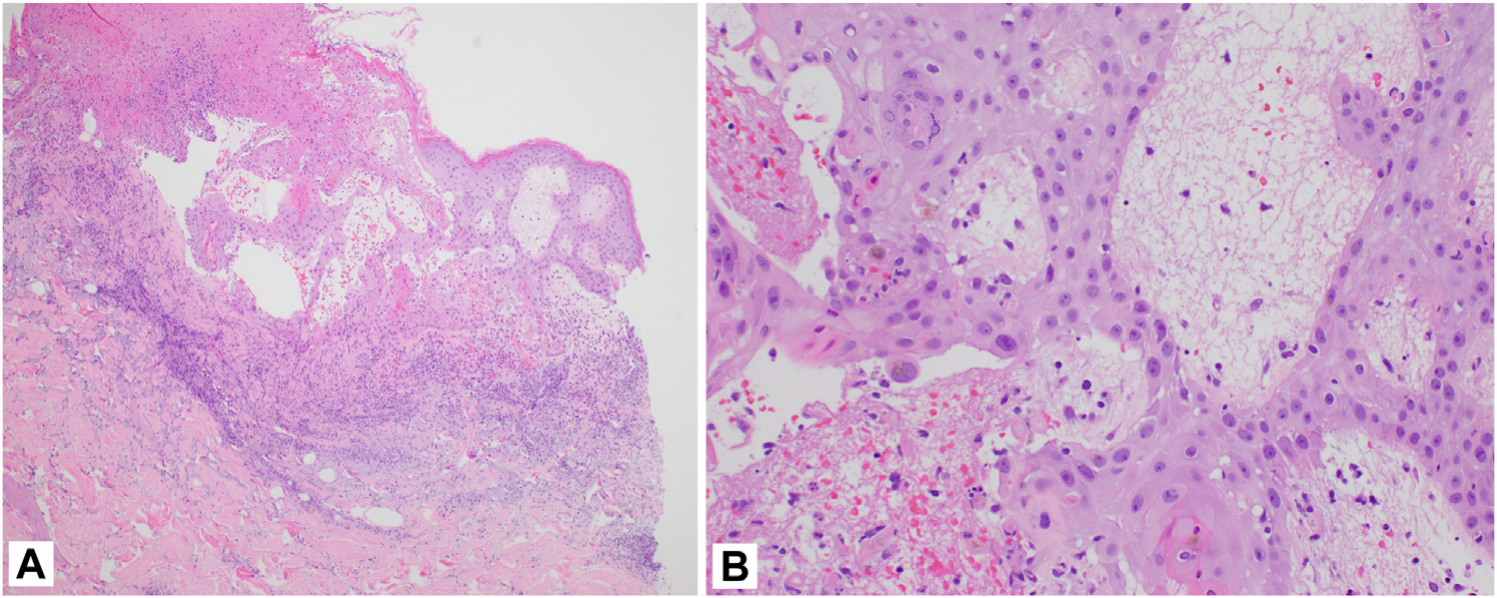
Case 1. A punch biopsy showing epidermal necrosis, vesiculation, and superficial lymphocytic infiltrate. (A and B, Hematoxylin-eosin stain; A, low power; B, high power.)

A 76-year-old man with American Joint Committee on Cancer Stage III C^3^ melanoma of the upper portion of the right arm was treated with 1.2 mL of 10^6^-plaque-forming units (PFU)/mL T-VEC divided among 4 in-transit lesions. On postinjection day (PID) 10, vesicles developed on the right side of the chest, right axilla, and right arm (Fig 1). Although both punch biopsies taken yielded negative results for HSV 1 and 2, as determined using immunohistochemistry, a diagnosis of HSV infection was made based on the pattern of inflammation and epidermal necrosis (Fig 2) and a positive result of polymerase chain reaction for vesicular fluid HSV. T-VEC was discontinued, and a 10-day course of valacyclovir resolved the rash.

## Case 2

**Fig 3:**
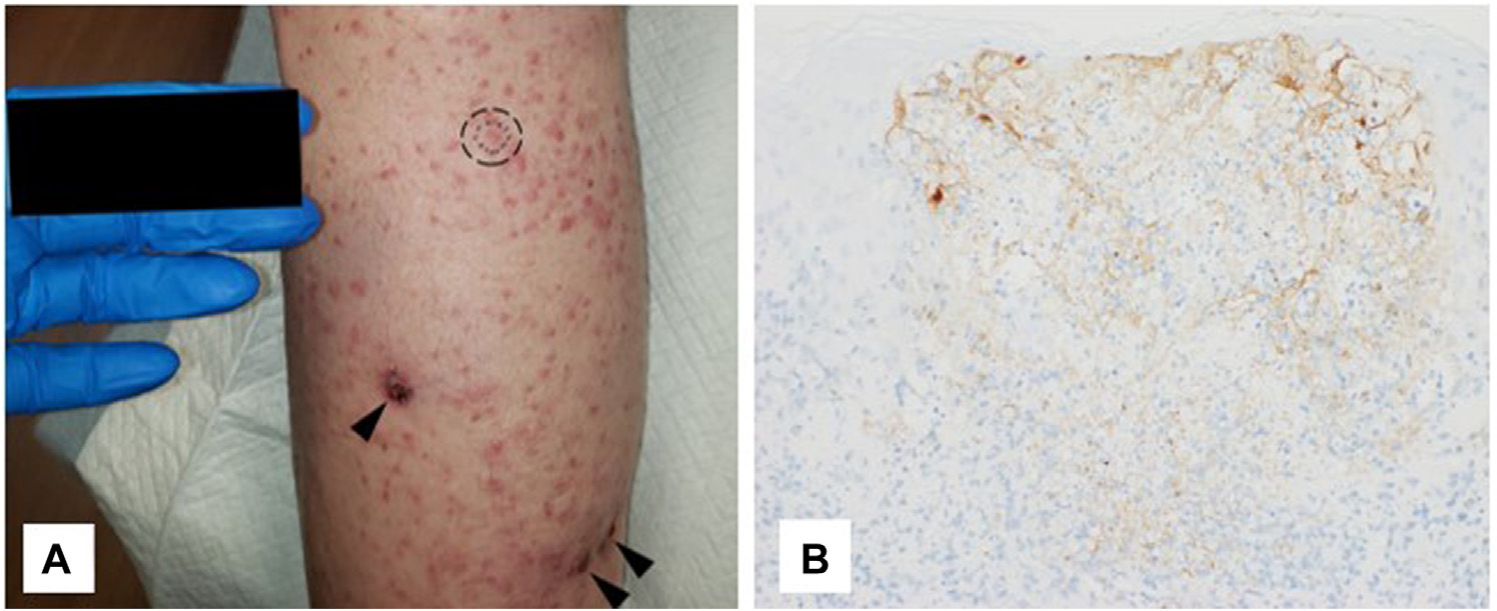
Case 2. A, The rash 8 days after talimogene laherparepvec injection. The *dashed ellipse* encompasses the punch biopsy site for the diagnosis of herpes simplex virus. *Arrowheads* designate the nodules of in-transit melanoma injected with talimogene laherparepvec. B, Positive herpes simplex virus 1/2 immunostaining in the vesicle demonstrated in the punch biopsy.

A 67-year-old man with stage IIID^3^ melanoma of the right heel was treated with 0.5 mL of 10^6^-PFU/mL T-VEC divided among 24 in-transit lesions. On PID 1, he developed fever, arthralgias, and fatigue. On PID 7, he developed a nontender, nonpruritic, vesicular rash extending beyond the injection sites. On PID 8 (Fig 3), 1 of 2 biopsied lesions was HSV positive, as determined using immunohistochemistry (Fig Y). T-VEC was discontinued, and a 10-day course of valacyclovir resolved the rash.

## Case 3

**Fig 4:**
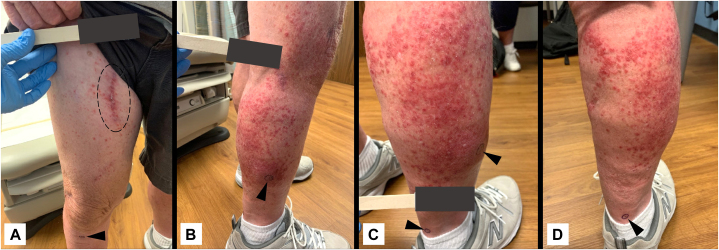
A, B, C, D, Case 3. The rash 8 days after talimogene laherparepvec injection. The *dashed ellipse* encompasses the surgical scar from the excision of the primary melanoma. The *arrowhead* designates the nodule of in-transit melanoma injected with talimogene laherparepvec.

A 79-year-old man with stage IIIC^3^ melanoma of the right plantar foot was treated with 0.3 mL of 10^6^-PFU/mL T-VEC divided among 3 in-transit lesions. On PID 3, he developed diffuse, mild erythema and fever, which progressed by PID 4 to a nonpruritic, nontender, and nonexudative violaceous eruption extending to the thigh. On PID 8, the patient presented with crusted, grouped erythematous papules (Fig 4); a punch biopsy yielded a positive result for HSV, as determined using immunohistochemistry. T-VEC was discontinued, and a 10-day course of valacyclovir resolved the rash.

## Case 4

A 73-year-old man with stage III^3^ melanoma of the left calf was treated with 0.5 mL of T-VEC at 10^6^ PFU/mL in a region of subcutaneous metastasis of the left thigh. Between PIDs 7 and 8, he reported arthralgias, myalgia, fever, generalized weakness, severe fatigue, dyspnea, an injection-site vesicular rash, altered mentation, and imbalance requiring admission to the intensive care unit. Empiric intravenous acyclovir and broad-spectrum antibiotics were initiated, and polymerase chain reaction test of cerebral spinal fluid ultimately returned a positive result for HSV-1. A 3-week course of intravenous acyclovir and physical therapy resolved all symptoms. T-VEC was discontinued.

## Conclusions

Our patients had no known immunocompromising factors or history of HSV infection and only received the initial 10^6^-PFU/mL dose of T-VEC. Two cases had received prednisone; however, T-VEC was initiated more than 1 month after prednisone discontinuation. Both the case described by Kimmis et al^2^ and our case had received prior immune checkpoint inhibitor treatment.^2^ Although it is possible that immune checkpoint inhibitors are associated with an increased risk of disseminated HSV, our patients had discontinued immune checkpoint inhibitors over 3 months prior to T-VEC treatment.

Our cases substantiate the risk of disseminated HSV in immunocompetent patients receiving T-VEC and demonstrate that immediate T-VEC cessation and antiviral therapy can truncate the disease course and lead to resolution, without permanent sequelae, even in the case of encephalitis.

## Conflicts of interest

Dr Zager has advisory board relationships and has received fees from with Merck, Novartis, Philogen, Castle Biosciences, Pfizer, and Sun Pharma. He also received research funding from Amgen, Delcath Systems, Philogen, Provectus, and Novartis. He serves on the medical advisory board for Delcath Systems. Dr Sarnaik is a coinventor on a patent application with Provectus Biopharmaceuticals. He has received ad hoc consulting fees from Iovance Biotherapeutics, Guidepoint, Defined Health, Huron Consulting Group, KeyQuest Health Inc, Istari, Rising Tide, and Gerson Lehrman Group. He has received speaker fees from the Society for Immunotherapy of Cancer, Physicians’ Educational Resource LLC, Medscape, and Medstar Health. The remaining authors have no relevant conflicts of interest to disclose.

## References

[1] Shmuylovich L., McEvoy A.M., Fields R.C., Hernandez-Aya L., Ansstas G., Chen D.Y. (2022). Durable melanoma control following disseminated talimogene laherparepvec herpetic infection. JAAD Case Rep.

[2] Kimmis B.D., Luu Y., Dai H. (2022). Disseminated herpes infection following talimogene laherparepvec administration. JAMA Dermatol.

[3] Amin M.B., Greene F.L., Byrd D.R. (2017). AJCC Cancer Staging Manual.

